# A phase II study of temozolomide vs. procarbazine in patients with glioblastoma multiforme at first relapse

**DOI:** 10.1054/bjoc.2000.1316

**Published:** 2000-08-16

**Authors:** W K A Yung, R E Albright, J Olson, R Fredericks, K Fink, M D Prados, M Brada, A Spence, R J Hohl, W Shapiro, M Glantz, H Greenberg, R G Selker, N A Vick, R Rampling, H Friedman, P Phillips, J Bruner, N Yue, D Osoba, S Zaknoen, V A Levin

**Affiliations:** Department of Neuro-Oncology, UTMD Anderson Cancer Center, Box 100, 1515 Holcombe Boulevard, Houston, Texas, 77030; Barret Cancer Center, 234 Goodman Street, Cincinnati, Ohio, 45267; Department of Neurosurgery, Emory University, 1327 Clifton Road, Atlanta, Georgia, 30322; University of Mississippi Medical Center, 2500 North State Street, Jackson, Mississippi, 39216; Department of Neurology, University of Texas, Southwestern Medical School, 5323 Harry Hines Boulevard, Dallas, Texas, 75235; Department of Neurosurgery, University of California San Francisco, 533 Parnassus Street, Room U107, San Francisco, CaliCityfornia, 94143; The Royal Marsden NHS Trust and the Institute of Cancer Research, Royal Marsden Hospital, Downs Road, Sutton, Surrey, SM2 5PT, United Kingdom; Department of Neurology, University of Washington, Box 356465, 1959 N.E. Pacific Street, Seattle, Washington, 98195; University of Iowa, Hospitals and Clinics, 200 Hawkins Drive, Iowa City, Iowa, 52242; Division of Neurology, Barrow Neurological Institute, 350 West Thomas Road, Phoenix, Arizona, 85013; Memorial Hospital of Rhode Island, 710 Robinson Road, PO Box 665, Hinsdale, Massachusetts, 01235; Department of Neurology, University of Michigan Medical Center, 1500 East Medical Center Drive, 1914 Taubman Center, Box 0316, Ann Arbor, Michigan, 48109; West Penn Hospital, Center for Neuro-Oncology, 4800 Friendship Avenue – Suite 1614, Pittsburgh, Pennsylvania, 15224; Division of Neurology, Evanston Hospital, 2650 Ridge Avenue, Evanston, Illinois, 60201; Beatson Oncology Centre, Western Infirmary, Glasgow, G11 6NT, United Kingdom; Department of Pediatric Hematology-Oncology, Duke University Medical Center, Duke North – Room 5418, Erwin Road, Durham, North Carolina, 27710; Department of Neuroscience, University of Pennsylvania Medical Center, Abramson 516, 3400 Civic Center Boulevard, Philadelphia, Pennsylvania, 19104; MRI Reading Center at St. Joseph, 8216 Carrbridge Circle, Baltimore, Maryland, 21204; British Columbia Cancer Agency, 1515 Larch Street, Vancouver, British Columbia, V6K 3N6, Canada; Schering-Plough Research Institute, 2000 Galloping Hill Road, Kenilworth, New Jersey, 07033, USA

**Keywords:** malignant glioma, temozolomide, procarbazine, glioblastoma multiforme

## Abstract

A randomized, multicentre, open-label, phase II study compared temozolomide (TMZ), an oral second-generation alkylating agent, and procarbazine (PCB) in 225 patients with glioblastoma multiforme at first relapse. Primary objectives were to determine progression-free survival (PFS) at 6 months and safety for TMZ and PCB in adult patients who failed conventional treatment. Secondary objectives were to assess overall survival and health-related quality of life (HRQL). TMZ was given orally at 200 mg/m^2^/day or 150 mg/m^2^/day (prior chemotherapy) for 5 days, repeated every 28 days. PCB was given orally at 150 mg/m^2^/day or 125 mg/m^2^/day (prior chemotherapy) for 28 days, repeated every 56 days. HRQL was assessed using the European Organization for Research and Treatment of Cancer Quality of Life Questionnaire (EORTC QLQ-C30 [+3]) and the Brain Cancer Module 20 (BCM20). The 6-month PFS rate for patients who received TMZ was 21%, which met the protocol objective. The 6-month PFS rate for those who received PCB was 8% (*P* = 0.008, for the comparison). Overall PFS significantly improved with TMZ, with a median PFS of 12.4 weeks in the TMZ group and 8.32 weeks in the PCB group (*P* = 0.0063). The 6-month overall survival rate for TMZ patients was 60% vs. 44% for PCB patients (*P* = 0.019). Freedom from disease progression was associated with maintenance of HRQL, regardless of treatment received. TMZ had an acceptable safety profile; most adverse events were mild or moderate in severity. © 2000 Cancer Research Campaign


*British Journal of Cancer* (2000) **83**(5), 588–593
© 2000 Cancer Research Campaign
doi: 10.1054/ bjoc.2000.1316, available online at http://www.idealibrary.com on IDEAL®

### A phase II study of temozolomide vs. procarbazine in patients with glioblastoma multiforme at first relapse

**WKA Yung[1], RE Albright[2], J Olson[3], R Fredericks[4], K Fink[5], MD Prados [6], M Brada[7], A Spence[8], RJ Hohl[9], W Shapiro[10], M Glantz[11], H Greenberg[12], RG Selker[13], NA Vick[14], R Rampling[15], H Friedman[16], P Phillips[17], J Bruner[1], N Yue[18], D Osoba[19], S Zaknoen[20] and VA Levin[1]**

[1]UTMD Anderson Cancer Center, Department of Neuro-Oncology, Box 100, 1515 Holcombe Boulevard, Houston, Texas 77030; [2]Barret Cancer Center, 234 Goodman Street, Cincinnati, Ohio 45267; [3]Emory University, Department of Neurosurgery, 1327 Clifton Road, Atlanta, Georgia 30322; [4]University of Mississippi Medical Center, 2500 North State Street, Jackson, Mississippi 39216; [5]University of Texas, Southwestern Medical School, Department of Neurology, 5323 Harry Hines Boulevard, Dallas, Texas 75235; [6]University of California, San Francisco, Department of Neurosurgery, 533 Parnassus Street, Room U107, San Francisco, California 94143; [7]The Royal Marsden NHS Trust and the Institute of Cancer Research, Royal Marsden Hospital, Downs Road, Sutton, Surrey, SM2 5PT, United Kingdom; [8]University of Washington, Department of Neurology, Box 356465, 1959 N.E. Pacific Street, Seattle, Washington 98195; [9]University of Iowa, Hospitals and Clinics, 200 Hawkins Drive, Iowa City, Iowa 52242; [10]Barrow Neurological Institute, Division of Neurology, 350 West Thomas Road, Phoenix, Arizona 85013; [11]Memorial Hospital of Rhode Island, 710 Robinson Road, PO Box 665, Hinsdale, Massachusetts 01235; [12]University of Michigan Medical Center, Department of Neurology, 1500 East Medical Center Drive, 1914 Taubman Center, Box 0316, Ann Arbor, Michigan 48109; [13]West Penn Hospital, Center for Neuro-Oncology, 4800 Friendship Avenue – Suite 1614, Pittsburgh, Pennsylvania 15224; [14]Evanston Hospital, Division of Neurology, 2650 Ridge Avenue, Evanston, Illinois 60201; [15]Beatson Oncology Centre, Western Infirmary, Glasgow G11 6NT, United Kingdom; [16]Duke University Medical Center, Department of Pediatric Hematology-Oncology, Duke North – Room 5418, Erwin Road, Durham, North Carolina 27710; [17]University of Pennsylvania Medical Center, Department of Neuroscience, Abramson 516, 3400 Civic Center Boulevard, Philadelphia, Pennsylvania 19104; [18]MRI Reading Center at St. Joseph, 8216 Carrbridge Circle, Baltimore, Maryland 21204; [19]British Columbia Cancer Agency, 1515 Larch Street, Vancouver, British Columbia, Canada V6K 3N6; [20]Schering-Plough Research Institute, 2000 Galloping Hill Road, Kenilworth, New Jersey 07033 USA

**Summary** A randomized, multicentre, open-label, phase II study compared temozolomide (TMZ), an oral second-generation alkylating agent, and procarbazine (PCB) in 225 patients with glioblastoma multiforme at first relapse. Primary objectives were to determine progression-free survival (PFS) at 6 months and safety for TMZ and PCB in adult patients who failed conventional treatment. Secondary objectives were to assess overall survival and health-related quality of life (HRQL). TMZ was given orally at 200 mg/m$^2$/day or 150 mg/m$^2$/day (prior chemotherapy) for 5 days, repeated every 28 days. PCB was given orally at 150 mg/m$^2$/day or 125 mg/m$^2$/day (prior chemotherapy) for 28 days, repeated every 56 days. HRQL was assessed using the European Organization for Research and Treatment of Cancer Quality of Life Questionnaire (EORTC QLQ-C30 [+3]) and the Brain Cancer Module 20 (BCM20). The 6-month PFS rate for patients who received TMZ was 21%, which met the protocol objective. The 6-month PFS rate for those who received PCB was 8% ($P$ = 0.008, for the comparison). Overall PFS significantly improved with TMZ, with a median PFS of 12.4 weeks in the TMZ group and 8.32 weeks in the PCB group ($P$ = 0.0063). The 6-month overall survival rate for TMZ patients was 60% vs. 44% for PCB patients ($P$ = 0.019). Freedom from disease progression was associated with maintenance of HRQL, regardless of treatment received. TMZ had an acceptable safety profile; most adverse events were mild or moderate in severity. © 2000 Cancer Research Campaign

**Keywords:** malignant glioma; temozolomide; procarbazine; glioblastoma multiforme

Glioblastoma multiforme (GBM) is the most common and malignant primary brain tumour (Chamberlain and Kormanik, 1998). Standard treatment for the initial treatment of these tumours is surgical resection and postoperative radiation. A meta-analysis of randomized clinical trials reports a modest survival advantage with adjuvant chemotherapy (Fine et al, 1993). However, a recent Medical Research Council randomized trial of 674 patients failed to show a survival benefit of nitrosourea-based adjuvant chemotherapy (Brada et al, 1998). Regardless of initial treatment, most patients relapse (Feun et al, 1994). Single-agent or combination salvage chemotherapy regimens for recurrent high-grade glioma remain palliative; median survival is 6 to 8 months, and few patients survive more than 2 years (Forsyth, 1996; Chamberlain and Kormarik 1998).

Temozolomide (TMZ) is a new orally administered, second-generation imidazotetrazine prodrug with essentially 100% oral bioavailability (Newlands et al, 1992). Preclinically, TMZ demonstrated broad spectrum, schedule dependent, antitumour activity with relatively little toxicity. TMZ spontaneously converts to the active alkylating agent 5-(3-methyltriazen-1-yl) imidazole-4-carboximide under physiologic conditions (Stevens et al, 1987; Tsang et al, 1990) with extensive tissue distribution, including penetration of the blood–brain barrier and the cerebrospinal fluid (Patel et al, 1995; Brock et al, 1996, 1997). In phase I and II clinical studies, TMZ was well tolerated with a favourable toxicity profile, with easily managed noncumulative myelotoxicity. TMZ has notable antitumour activity against recurrent GBM, recurrent anaplastic astrocytoma, and advanced malignant melanoma, and

*Received 13 August 1999*
*Revised 23 April 2000*
*Accepted 1 May 2000*

*Correspondence to:* WKA Yung other refractory cancers (Newlands et al, 1992, 1996; O'Reilly et al, 1993; Bleehen et al, 1995; Bower et al, 1997).

This multicentre phase II study in patients with GBM at first relapse was prompted by the activity of TMZ in recurrent or progressive high-grade glioma and the need for new single-agent treatments with improved efficacy, safety and health-related quality of life (HRQL). The primary objectives were to evaluate the progression-free survival (PFS) at 6 months and safety of TMZ and the reference agent, procarbazine (PCB). PCB is one of the few orally administered therapeutic options available to patients with high-grade gliomas, particularly those who fail radiation and nitrosourea therapy (Kumar et al, 1974; Rodriguez et al, 1989; Newton et al, 1990). The secondary objectives were to evaluate the effect of TMZ on overall survival, HRQL and response rate.

### PATIENTS AND METHODS

### Patients

Eligible patients were ≥18 years old, had histologically proven supratentorial GBM or gliosarcoma and unequivocal evidence of tumour recurrence or progression at first relapse by gadolinium magnetic resonance imaging (Gd-MRI) or by contrast-enhanced computerized tomography (CT) scanning after radiation therapy for initial disease. Patients could have had one course of chemotherapy that must have contained a nitrosourea and were required to have a Karnofsky performance status (KPS) of ≥70 and a life expectancy of ≥12 weeks at study entry.

Patients excluded had evidence of significant renal, hepatic or bone marrow impairment (i.e. blood urea nitrogen and creatinine <1.5 times the upper limit of normal; total and direct serum bilirubin <1.5 times the upper limit of normal; absolute neutrophil count [ANC] ≥1500/µl; platelet count >100 000/µl; and haemoglobin >10 g/dl). Other exclusion criteria were >1 prior chemotherapy regimen; history of previous chemotherapy with single-agent PCB or dacarbazine; a regimen of chemotherapy (excluding vincristine, nitrosourea or mitomycin C) within 4 weeks before study drug administration; vincristine within 2 weeks before study drug administration; nitrosourea or mitomycin C within 6 weeks before study drug administration or a history of PCB-induced rash; previous interstitial radiotherapy or stereotactic radiosurgery; pregnancy; breast feeding. Women of childbearing potential had to be using adequate contraceptive precautions. Patients experiencing toxicities from prior therapy, those with medical conditions (HIV) and those with previous or concurrent solid tumours at other sites (excluding basal cell carcinomas) were also excluded.

### Study design and drug administration

The protocol was approved by the institutional review board or independent ethics committee of each investigative facility and conducted according to the Declaration of Helsinki and its amendments. Patients gave informed consent before entering the study. A blinded centralized review of neuropathology and neuroradiology was performed. Eligibility was determined by blinded centralized review of at least one histologic specimen from each surgical procedure. GBM was classified according to the Burger and Nelson system (Burger et al, 1985; McComb and Barger, 1985).

Monthly performance and clinical evaluation, neurologic examinations, and quality-of-life measurements (questionnaire) were done. Tumour was evaluated every 2 months with Gd-MRI or contrast-enhanced CT scan. Image-based evidence of progression is detectable before clinical deterioration and PFS was determined by image-based progression on Gd-MRI scans, considering all neurologic and clinical data indicative of disease progression. Comprehensive evaluation of neurologic status was performed at study visits comparing signs and symptoms from previous examination. Grading was definitely better (+2); possibly better (+1); unchanged (0); possibly worse (–1); or definitely worse (–2).

Baseline Gd-MRI brain scan must have documented evaluable (measurable or nonmeasurable) residual disease <14 days before study drug administration. Patients were required to be on stable doses of steroids for ≥7 days before the Gd-MRI scan. A baseline Gd-MRI scan was performed within 72 h or 8 to 12 weeks after surgical resection in patients undergoing surgery at first relapse.

#### *TMZ dosing*

TMZ was administered orally at a dosage of 150 mg/m$^2$/day (750 mg/m$^2$ total dose per cycle) on days 1–5 to fasting patients who had received previous chemotherapy or at a dosage of 200 mg/m$^2$/day (1000 mg/m$^2$ total dose per cycle) for patients who had not received previous chemotherapy. Treatment cycles were repeated every 28 days.

#### *PCB dosing*

PCB was administered orally at a starting dosage of 125 mg/m$^2$/day for 28 consecutive days (days 1–28) to those who had received previous chemotherapy or at a dosage of 150 mg/m$^2$/day to patients who had not received previous chemotherapy. Treatment cycles were repeated every 56 days.

#### *Retreatment criteria*

If the ANC was ≥2000/µl and the platelet count was ≥125 000/µl, repeat cycles could be administered to a maximum dose of TMZ 200 mg/m$^2$/day or PCB 220 mg/m$^2$/day. In patients with nadir ANC of 500–999 mm$^3$ and nadir platelet count of 25,000–49,000 mm$^3$, TMZ was reduced to the next lower dosage level (150 mg/m$^2$/day or 100 mg/m$^2$/day), and PCB dose was reduced by 25% from that of the previous cycle. In patients with nadir ANC of <500 mm$^3$ and nadir platelet count of <25 000 mm$^3$, TMZ was reduced to the next lower dosage level, and PCB was reduced by 50% from that of the previous cycle. For grades 3 and 4 nonhaematologic toxicity, TMZ was reduced by two dosage levels, and PCB was reduced by 50% from that of the previous cycle. Patients continued treatment until unacceptable toxicity developed, disease progressed or 2-year treatment was completed.

#### *Other medications*

Patients continued on the lowest steroid dosage necessary for neurologic stability. Colony-stimulating factors were used only in cases of grade 4 neutropenia, when granulocyte colony-stimulating factor was permitted.

#### *Response determination*

A complete response (CR) was the disappearance of all enhancing tumour on consecutive MRI scans >1 month apart; discontinuation of steroids (except for physiologic doses that may have been

© 2000 Cancer Research Campaign required after prolonged therapy); and stable or improved neurologic status. Partial response (PR) was defined as ≥50% reduction in the sum of the products of the largest perpendicular diameters of contrast enhancement for all measurable lesions or an assessment of ‘definitely better’ for all nonmeasurable lesions on consecutive MRI scans at least 1 month apart; stable steroid use for 7 days before each scan (at dosage ≤ than had been administered at the time of the previous scan); neurologic status stable or improved. Progressive disease was a ≥25% increase in size of the product of the largest perpendicular diameters of contrast enhancement for any measurable lesions or an assessment of ‘definitely worse’ for any nonmeasurable lesions or any new tumour on MRI scans. All other situations were considered stable disease (SD).

### *Adverse drug-mediated events*

Adverse events (AEs) were tabulated according to treatment group. Treatment-emergent AEs were those beginning during or within 30 days after the end of treatment or that began before and worsened during treatment, regardless of any relationship to treatment. If CTC criteria were not defined for a parameter, the following grading scale was used: 0 = none; 1 = mild; 2 = moderate; 3 = severe; 4 = life-threatening. Abnormal laboratory values were recorded as AEs if they resulted in hospitalisation, transfusion of blood products or discontinuation of therapy. Worsening neurologic deficit was considered as a physical finding associated with neurologic examination rather than an AE.

### *HRQL*

The European Organization for Research and Treatment of Cancer Quality of Life Questionnaire (EORTC-QLQ-C30 [+3]) (Aaronson et al, 1993; Osoba et al, 1997) and the Brain Cancer Module 20 (BCM20) (Osoba et al, 1996) questionnaires were used to measure HRQL. Questionnaires were administered on day 1 of cycle 1 and at every visit throughout the study. Seven HRQL domains were selected a priori as most clinically relevant to patients with brain cancer: role, social functioning and global quality of life (from the EORTC-QLQ-C30 [+3]) and visual disorder, motor dysfunction, communication deficit and drowsiness (from the BCM20).

HRQL domains were scored according to EORTC instructions (Fayers et al, 1995). Tests of statistical significance were not applied. Meaning of changes in HRQL scores was addressed by the method of Osoba et al (1998). A 10-unit change on a 0–100 scale was deemed significant if it lasted for at least 8 weeks (Jaeschke et al, 1989; Juniper et al, 1994; King, 1996; Ware et al, 1998). Changes in HRQL scores from baseline were calculated for patients at the same time points as HRQL completion. Proportions of patients achieving an HRQL response (i.e. a 10-point improvement from baseline in a domain for at least 2 consecutive months) were calculated for each group.

### *Statistical methods*

The primary objective was estimation of PFS at 6 months in the intent-to-treat (ITT) population. With 100 patients per group, assuming that the true 6-month PFS rate for TMZ was 20%, the 95% confidence interval (CI) would range from 12.2–27.8%. This assured with confidence that the lower boundary of the 95% CI for the 6-month PFS rate for TMZ would remain higher than 10%, which was assumed to be the threshold of effectiveness.

A log-rank test used retrospectively determined whether meaningful differences were detectable between the two groups. With only progressive disease and death as events, PFS was measured from the start date of treatment to an event date or the last evaluation. Overall survival was measured from the start date of treatment to the date of death or the last evaluation. The product-limit method (Kaplan-Meier) was used to estimate PFS and overall survival. A Cox regression model was used to assess influence of baseline characteristics on the effect of treatment for PFS and overall survival. Subgroup analyses for PFS and overall survival were done according to prognostic variables in the Cox model.

### RESULTS

### Patient characteristics

Between January 5, 1995, and October 28, 1997, 225 patients were randomised and became the ITT population. Five patients (TMZ, $n = 2$; PCB, $n = 3$) were randomized but not treated. Fifteen (13%) TMZ patients and 31 (27%) PCB patients discontinued the study for the following reasons other than disease progression: AEs (TMZ, 3%; PCB, 10%); death (TMZ, 3%; PCB, 5%); noncompliance with either dosing or visit schedule (TMZ, 0%; PCB, 4%); failure to meet protocol eligibility (no treatment) (TMZ, 2%; PCB, 1%); completed treatment (TMZ, 2%; PCB, 1%) or did not wish to continue (TMZ, 4%; PCB, 4%). Histologic review indicated that tumours in 102/112 (91%) TMZ-treated patients and 108/113 (96%) PCB-treated patients were classified correctly as GBM or gliosarcoma. Most patients excluded after reclassification were judged to have anaplastic astrocytoma (7) or anaplastic oligoastrocytoma (3). Specimens for two patients were not reviewed, and one sample was inadequate for review. Baseline and disease characteristics were similar between treatment groups ($P \geq 0.19$) (Table 1). Median time from the end of radiation therapy to first relapse and median time from initial diagnosis to first relapse were

**Table 1** Demographics of ITT population

| | TMZ (*n* = 112) | PCB (*n* = 113) | *P* value |
|---|---|---|---|
| Age (years) | | | 0.43[a] |
| Median | 52 | 51 | |
| Range | 21–76 | 21–74 | |
| Sex | | | 0.48[b] |
| Male | 77 (69%) | 72 (64%) | |
| Race | | | 0.20[c] |
| White | 106 (95%) | 99 (88%) | |
| Black | 4 (4%) | 7 (6%) | |
| Other | 2 (2%) | 7 (6%) | |
| KPS | | | 0.19[c] |
| 100 | 3 (3%) | 10 (9%) | |
| 90 | 32 (29%) | 30 (27%) | |
| 80 | 43 (38%) | 34 (30%) | |
| 70 | 34 (30%) | 38 (34%) | |
| Not recorded | 0 | 1 (1%) | |
| Prior therapy | | | |
| Surgical resection at initial diagnosis | 87% | 91% | |
| Adjuvant nitrosourea-based chemotherapy | 65% | 68% | |
| Time from initial diagnosis to first relapse (mo) | 7 (3.1–66) | 8.4 (2.2–92.3) | 0.02[a] |
| Time from end radiotherapy to first relapse (months) | 4.6 (0.6–63.6) | 5.8 (0.1–92.3) | 0.03[a] |

[a]Kruskal-Wallis test; [b]Fisher’s exact test; [c]Chi-square test

 © 2000 Cancer Research Campaign

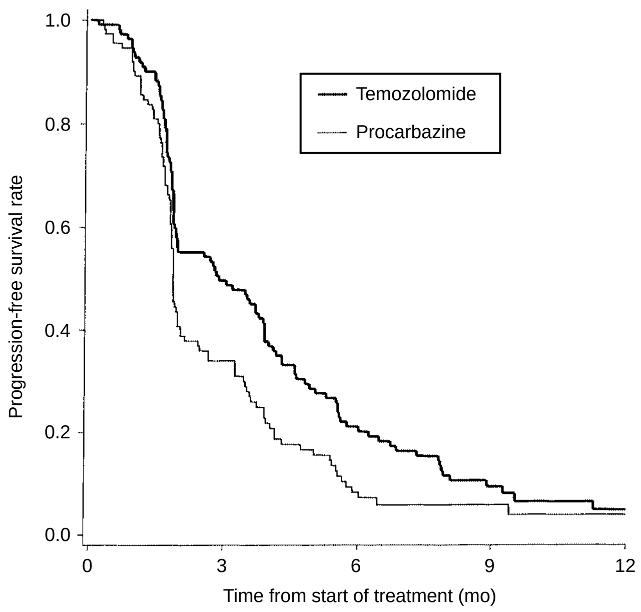

**Figure 1** PFS: ITT population

Reproduced by kind permission of W. B. Saunders Company from Yung WKA (2000) Temozolomiole in malignant gliomas. *Semin Oncol* **27** (3 Suppl. 6): 27–34

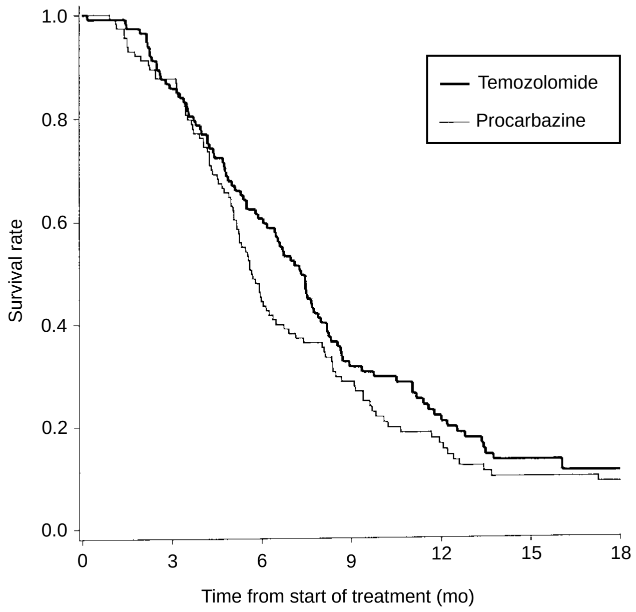

**Figure 2** Overall survival: ITT population

Reproduced by kind permission of W. B. Saunders Company from Yung WKA (2000) Temozolomiole in malignant gliomas. *Semin Oncol* **27** (3 Suppl. 6): 27–34

shorter for TMZ-treated patients, but Cox regression analysis revealed that these differences did not affect the overall inferences of the study.

### *Six-month PFS*

For TMZ patients, the 6-month PFS rate was 21% (95% CI, 13–29%), compared with 8% for PCB (95% CI, 3–14%; hazard ratio, $n$ = 1.54; $P$ = 0.008). The 21% 6-month PFS for TMZ met the protocol objective. Respective median PFS was 12.4 weeks for TMZ patients and 8.32 weeks for PCB patients. The overall progression-free interval also favoured TMZ ($P$ = 0.0063) with a hazard ratio of 1.47 (95% CI, 1.11–1.95%) (Figure 1). The differences in PFS rate appeared early and were maintained for several months. Results in the subgroup with eligible histology were similar to those reported for the ITT population. In the eligible population, the 6-month PFS rate was 19% (95% CI, 11–27%), compared with 9% for PCB (95% CI, 3–14%) The overall progression-free interval also favoured TMZ ($P$ = 0.0063) in the eligible population with a hazard ratio of 1.37. Analysis of data from time of randomisation showed similar results, with a 6-month PFS rate of 21% (95% CI, 13–29%), compared with 9% for PCB (95% CI, 4–15%; $P$ = 0.016).

### *Overall survival*

The median overall survival with TMZ was 1.5 months longer than that with PCB, but this difference did not reach statistical significance ($P$ = 0.330). At 6 months, survival of the TMZ-treated patients was 60% (95% CI, 51–70%) compared with 44% in PCB-treated patients (95% CI, 35–53%; hazard ratio = 1.44; $P$ = 0.019). The overall survival advantage for TMZ appeared at 4–6 months and persisted to at least 8 months (Figure 2). Analysis from time of randomisation showed similar results in median overall survival.

### *Prognostic factors and subgroup analyses*

Multivariate analysis of baseline characteristics indicated that PFS was related to baseline KPS ($P$ = 0.0107), and overall survival was related to baseline KPS ($P$ = 0.0024) and age ($P$ = 0.0359). Patients were stratified by age (>50 or ≤50 years), time from initial diagnosis to first relapse (>8 or ≤8 months), time from end of radiation therapy at initial diagnosis to first relapse (>6 or ≤6 months) and baseline KPS (>80 or ≤80). Analyses of these subgroups demonstrated a consistent advantage for TMZ over PCB on PFS and overall survival regardless of the subgroup; the hazard ratio always exceeded 1.

### *Objective responses*

According to the central reviewer's assessment of objective response, a PR occurred in 6/112 (5.4%) TMZ patients and in 6/113 (5.3%) PCB patients. SD occurred in 45/112 (40.2%) TMZ patients and 31/113 (27.4%) PCB patients. Overall response rates (PR + SD) of 51/112 (45.6%) in the TMZ group were significantly higher than those of 37/113 (32.7%) in PCB group ($P$ = 0.049).

### *HRQL assessment*

HRQL scores were available at baseline and postbaseline for 179/220 (81.4%) treated patients. Regardless of the treatment, HRQL scores were maintained at baseline levels prior to disease progression but then decreased substantially at the time of disease progression in six of the seven domains considered most clinically relevant to patients (data not shown). Across all seven domains, the proportions of patients achieving an HRQL response were consistently higher for TMZ than they were for PCB (Figure 3).

### *Toxicity*

Safety was assessed in the ITT population. Five patients (TMZ, $n$ = 2; PCB, $n$ = 3) who did not receive at least one dose of study

*© 2000 Cancer Research Campaign*

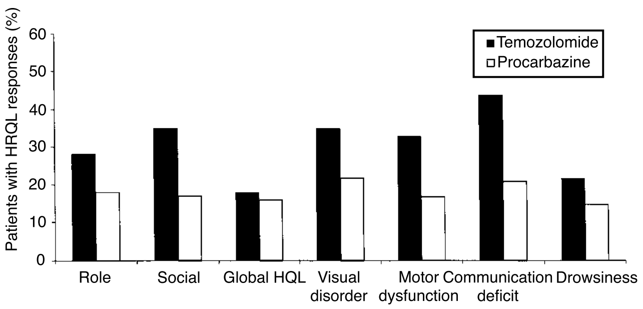

**Figure 3** Frequency of HRQL responses (seven domains)

Reproduced by kind permission of W. B. Saunders Company from Yung WKA (2000) Temozolomiole in malignant gliomas. *Semin Oncol* **27** (3 Suppl. 6): 27–34

medication were excluded from the analysis. A total of 110 TMZ-treated patients received 484 cycles of TMZ, and 110 PCB-treated patients received 167 cycles. Most PCB recipients were not treated beyond cycle 1. By the end of week 12, 62/110 (56%) TMZ patients and 36/110 (30%) PCB patients remained in the study, others dropping out primarily for disease progression or toxicity.

TMZ and PCB were generally well tolerated, with similar incidence of AEs in both groups (Table 2). The incidence of any AE was 77% in the TMZ group and 76% in the PCB group. As shown in Table 2, the incidence of grade 3 or 4 treatment-related AEs was less with TMZ (18%) than with PCB (25%). Serious AEs were reported for 169 patients (TMZ, $n$ = 87; PCB, $n$ = 82), primarily for hospitalizations due to disease progression.

TMZ-associated myelosuppression was predictable and reversible with no evidence of cumulative myelotoxicity. Nadir platelet counts or ANCs typically occurred around days 21–28 and recovered generally within 2 weeks. Most frequent AEs with TMZ were nausea (42%), vomiting (35%) and fatigue (30%). Nausea and vomiting were mild to moderate in most cases and were either self-limiting or controlled with antiemetics. Most common CTC grade 3 or 4 toxicities were thrombocytopenia (7%), neutropenia (4%), fatigue (3%), vomiting (3%) and nausea (3%) in a small number of patients in both treatment groups.

### DISCUSSION

This large trial involving 255 patients demonstrates the efficacy and safety of TMZ in the treatment of patients with recurrent GBM. This confirms the results of several phase II studies of TMZ in high-grade gliomas (Newlands et al, 1992, 1996; O'Reilly et al, 1993; Bower et al, 1997). Although the median time from initial diagnosis to first relapse as well as the median time from the end of radiation therapy to first relapse was shorter for TMZ recipients than for PCB, a Cox regression analysis showed that these differences did not affect study outcome.

To ensure an objective index of efficacy, Gd-MRI and a mandatory central review were used to evaluate response. Because steroid therapy can produce changes in imaging studies of central nervous system malignancies, a stable dose of steroids before the initial Gd-MRI scan was required; overall response criteria specified that use of steroids should be stable for ≥7 days before the investigator's assessment. Patients adhered to mandated schedules of medical imaging and neurologic examination, and protocol variations were minimal. Oral PCB has demonstrated activity in relapsing malignant

**Table 2** Treatment-related AEs reported in ≥2% of patients in either treatment group during days 1–56, safety population

| | Number (%) of patients[a] | | | |
|---|---|---|---|---|
| | TMZ ($n$ = 110) | | PCB ($n$ = 110) | |
| AEs | All | Grades 3/4 | All | Grades 3/4 |
| Any | 85 (77) | 20 (18) | 87 (76) | 28 (25) |
| Nausea | 42 (38) | 3 (3) | 37 (34) | 3 (3) |
| Vomiting | 35 (32) | 3 (3) | 30 (27) | 5 (5) |
| Fatigue | 30 (27) | 3 (3) | 16 (15) | 2 (2) |
| Constipation | 17 (15) | 1 (1) | 11 (10) | 1 (1) |
| Anorexia | 12 (11) | 0 | 9 (8) | 2 (2) |
| Headache | 13 (12) | 2 (2) | 9 (8) | 2 (2) |
| Rash | 7 (6) | 0 | 9 (8) | 1 (1) |
| Thrombocytopenia | 9 (8) | 8 (7) | 4 (4) | 4 (4) |
| Neutropenia | 4 (4) | 4 (4) | 4 (4) | 3 (3) |
| Anemia | 2 (2) | 1 (1) | 2 (2) | 2 (2) |
| Leukopenia | 2 (2) | 1 (1) | 0 | 0 |
| Diarrhea | 6 (5) | 0 | 8 (7) | 1 (1) |

[a]Haematologic AEs are included regardless of percentage gliomas. As the reference agent, PCB verified that objective end points could be seen within the definition of the protocol and permitted comparison of end points. The study size was designed to demonstrate equivalent efficacy between the two drugs. Overall response CPR+SD seen in response to PCB in this study (32.7%) were similar to that found by Rodriguez et al 1989), but less than that reported by Newton et al (1990). Comparisons between studies are difficult because of differences in pathologic evaluation of tumours and in the modalities used to measure disease progression.

PFS at 6 months, the primary end point of the protocol, was significantly longer with TMZ than with PCB (21% vs. 8%); overall PFS was also significantly longer for TMZ than for PCB. The difference in PFS in favour of TMZ was observed as early as 1 month after randomisation and was maintained for several months. Although the gain in PFS for TMZ patients did reflect longer overall survival, it did not translate into a significant gain in overall survival. PFS is a reliable and measurable end point when appropriate imaging modalities are used for tumour evaluation and is thus a clinically significant end point. Malignant gliomas are progressive and ultimately fatal tumours; treatment maintaining HRQL and useful survival for months is an important benefit. PFS is also not influenced by subsequent therapies that may follow progression.

Progressive, recurrent GBM causes deterioration of the patient's neurologic status and HRQL. A primary therapy goal in this incurable malignancy is symptom palliation and improvement or maintenance of HRQL. This study demonstrates that disease progression is associated with HRQL deterioration and delaying disease progression provides meaningful maintenance of HRQL to patients with brain tumours. Some of the differences in HRQL response between TMZ and PCB may have been related to the dosing of TMZ over 5 days as compared with PCB, which was dosed over a 28-day period.

Temozolomide has an acceptable safety profile in patients with GBM. Since most PCB recipients were not treated beyond cycle 1, patients received TMZ for a greater number of cycles. The number of patients reporting at least one treatment-related AE was similar for TMZ (77%) and PCB (76%), even though 90% of TMZ patients received more than one cycle of treatment, compared with only 33% of those treated with PCB. To provide balance, AEs

 *© 2000 Cancer Research Campaign*

were also examined for the first 56 days of treatment (i.e. one complete cycle for PCB). During this period, more PCB patients (21%) reported severe or life-threatening treatment-related AEs than TMZ patients (15%). Only three TMZ patients dropped out because of AEs compared with 11 PCB patients.

Although myelosuppression is a well-established dose-limiting side effect of treatment with all alkylating agents, it occurred in only a small number of TMZ recipients. When neutropenia and thrombocytopenia developed, they usually occurred in the first few treatment cycles, resolved with a one-level reduction in dosage and were not cumulative.

This study demonstrates the safety and efficacy of TMZ in recurrent GBM. TMZ is associated with an improvement in the patient's HRQL. The antitumour activity and predictability of TMZ may prove valuable in patients with anaplastic astrocytomas and in trials of patients with metastatic malignant melanoma and other therapy-resistant tumours. Further benefit is anticipated on combination of TMZ with agents such as the nitrosoureas, taxanes, topoisomerase inhibitors and biologic-response modifiers and in alternate dosing regimens (e.g. continuous-dosing studies).

### ACKNOWLEDGEMENTS

The authors would like to thank the following: William Spiro MD, Allen L. Cohn MD, Steven Rosenfeld MD, PhD, L. Austin Doyle MD, James Wilson MD, Hendricks Krouwer MD and Edward Dropcho MD

### REFERENCES

Aaronson NK, Ahmedzai S, Bergman B, Bullinger M, Cull A, Duez NJ, Filiberti A, Fletchner H, Fleishman SB and de Haes JC (1993) The European Organization for Research and Treatment of Cancer QLQ-C30: a quality-of-life instrument for use in international clinical trials in oncology. *J Natl Cancer Inst* **85**: 365–376

Bleehen NM, Newlands ES, Lee SM, Thatcher N, Selby P, Calvert AH, Rustin GJS, Brampton M and Stevens MFG (1995) Cancer research campaign phase II trial of temozolomide in metastatic melanoma. *J Clin Oncol* **13**: 910–913

Bower M, Newlands ES, Bleehen NM, Brada M, Begent RJH, Calvert H, Colquhoun I, Lewis P and Brampton MH (1997) Multicentre CRC phase II trial of temozolomide in recurrent or progressive high-grade glioma. *Cancer Chemother Pharmacol* **40**: 484–488

Brada M, Thomas DGT, Bleehen NM, Roberts JT, Senanayake F, Abram P, Lantos PL, Moss TH, Ironside JW, Whaley JB and Stenning SP (1998) *Proceedings of the American Society of Clinical Oncology* **17**: [Abstract 1543]

Brock CS, Matthews JC, Brown G, Luthra SK, Brady F, Newlands ES and Price P (1996) The kinetic behaviour of temozolomide in man. *Proceedings of the American Society of Clinical Oncology* **15**: 475 [Abstract 1502]

Brock CS, Matthews JC, Brown G, Newlands ES and Price P (1997) In vivo demonstration of $^{11}$C-temozolomide uptake by human recurrent high grade astrocytomas. *Br J Cancer* **75**: 1241 [Abstract]

Burger PC, Vogel FS, Green SB and Strike TA (1985) Glioblastoma multiforme and anaplastic astrocytoma. Pathologic criteria and prognostic implications. *Cancer* **56**: 1106–1111

Chamberlain MC and Kormanik PA (1998) Practical guidelines for the treatment of malignant gliomas. *West J Med* **168**: 114–120

Fayers P, Aaronson N, Bjordal K and Sullivan M (1995) *EORTC QLQ-C30 Scoring Manual*. Brussels, Belgium, Quality of Life Unit, EORTC Data Centre

Fine HA, Dear KB, Loeffler JS, Black PM and Canellos GP (1993) Meta-analysis of radiation therapy with and without adjuvant chemotherapy for malignant glioma in adults. *Cancer* **71**: 2585–2597

Feun LG, Savaraj N and Landy HJ (1994) Drug resistance in brain tumors. *J Neurooncol* **20**: 165–176

Forsyth PA and Cairncross JG (1996) Chemotherapy for malignant gliomas. *Baillières Clin Neurol* **5**: 371–393

Jaeschke R, Singer J and Guyatt GH (1989) Measurement of health status. Ascertaining the minimal clinically important difference. *Control Clin Trials* **10**: 407–415

Juniper EF, Guyatt GH, Willan A and Griffith LE (1994) Determining a minimal important change in a disease-specific quality of life questionnaire. *J Clin Epidemiol* **47**: 81–87

King MT (1996) The interpretation of scores from the EORTC quality of life questionnaire QLQ-C30. *Qual Life Res* **5**: 555–567

Kumar ARV, Renaudin J, Wilson CB, Boldrey EB, Enot KJ and Levin VA (1974) Procarbazine hydrochloride in the treatment of brain tumors. *J Neurosurg* **40**: 365–371

Levin VA, Leibel SA and Gutin PH (1997) Neoplasms of the central nervous system. In: DeVita Jr VT, Hellman S, Rosenberg SA, (eds). *Cancer Principals and Practice of Oncology*. 5th ed. Philadelphia: Lippincott Raven: 2041–2042

McComb RD and Burger PC (1985) Pathologic analysis of primary brain tumors. *Neurol Clin* **3**: 711–728

Newlands ES, Blackledge GRP, Slack JA, Rustin GJS, Smith DB, Stuart NSA, Quarterman CP, Hoffman R, Stevens MFG, Brampton MH and Gibson AC (1992) Phase I trial of temozolomide (CCRG 81045: M&B 39831: NSC 362856). *Br J Cancer* **65**: 287–291

Newlands ES, O'Reilly SM, Glaser MG, Bower M, Evans H, Brock C, Brampton MH, Colquhoun I, Lewis P, Rice-Edwards JM, Illingworth RD and Richards PG (1996) The Charing Cross Hospital experience with temozolomide in patients with gliomas. *Eur J Cancer* **32A**: 2236–2241

Newton HB, Junck L, Bromberg J, Page MA and Greenberg HS (1990) Procarbazine chemotherapy in the treatment of recurrent malignant astrocytomas after radiation and nitrosourea failure. *Neurology* **40**: 1743–1746

O'Reilly SM, Newlands ES, Glaser MG, Brampton M, Rice-Edwards JM, Illingworth RD, Richards PG, Kennard C, Colquhoun IR, Lewis P and Stevens MFG (1993) Temozolomide: a new oral cytotoxic chemotherapeutic agent with promising activity against primary brain tumours. *Eur J Cancer* **29A**: 940–942

Osoba D, Aaronson NK, Muller M, Sneeuw K, Hsu M-A, Yung WKA, Brada M and Newlands E (1996) The development and psychometric validation of a brain cancer quality-of-life questionnaire for use in combination with general cancer-specific questionnaires. *Qual Life Res* **5**: 139–150

Osoba D, Aaronson N, Zee B, Sprangers M and te Velde A (1997) Modification of the EORTC QLQ-C30 (version 2.0) based on content validity and reliability testing in large samples of patients with cancer. *Qual Life Res* **6**: 103–108

Osoba D, Rodrigues G, Myles J, Zee B and Pater J (1998) Interpreting the significance of changes in health-related quality-of-life scores. *J Clin Oncol* **16**: 139–144

Patel M, McCully C, Godwin K and Balis F (1995) Plasma and cerebrospinal fluid pharmacokinetics of temozolomide. *Proceedings of the American Society of Clinical Oncology* **14**: 461 [Abstract 1485]

Rodriguez LA, Prados M, Silver P and Levin VA (1989) Reevaluation of procarbazine for the treatment of recurrent malignant central nervous system tumours. *Cancer* **64**: 2420–2423

Stevens MFG, Hickman JA, Langdon SP, Chubb D, Vickers L, Stone R, Baig G, Goddard C, Gibson NW, Slack JA, Newton C, Lunt E, Fizames C and Lavelle F (1987) Antitumor activity and pharmacokinetics in mice of 8-Carbamoyl-3-methylimidazo [5,1-*d*]-1,2,3,5-tetrazin-4(3*H*)-one (CCRG 81045; M & B 39831), a novel drug with potential as an alternative to dacarbazine. *Cancer Res* **47**: 5846–5852

Tsang LLH, Farmer PB, Gescher A and Slack JA (1990) Characterisation of urinary metabolites of temozolomide in humans and mice and evaluation of their cytotoxicity. *Cancer Chemother Pharmacol* **26**: 429–436

Ware JE Jr, Kosinski M and Keller SD (1998-copyright) *SF-36 Physical and Mental Health Summary Scales: A User's Manual* [Homepage of QualityMetric], [Online]. Available: http://www.qmetric.com/products/manuals/SF-36-Summary.php3 [1999, March 1]

*© 2000 Cancer Research Campaign*
